# Exploring Nanocarriers as Treatment Modalities for Skin Cancer

**DOI:** 10.3390/molecules28155905

**Published:** 2023-08-05

**Authors:** Mohammad Adnan, Md. Habban Akhter, Obaid Afzal, Abdulmalik S. A. Altamimi, Irfan Ahmad, Manal A. Alossaimi, Mariusz Jaremko, Abdul-Hamid Emwas, Tanweer Haider, Md. Faheem Haider

**Affiliations:** 1 Faculty of Pharmacy, Integral University, Lucknow 226026, Uttar Pradesh, India; siddiquiadna856@gmail.com; 2 School of Pharmaceutical and Population Health Informatics (SoPPHI), DIT University, Dehradun 248009, Uttarakhand, India; habban.akhter@dituniversity.edu.in; 3 Department of Pharmaceutical Chemistry, College of Pharmacy, Prince Sattam Bin Abdulaziz University, Al-Kharj 11942, Saudi Arabia; o.akram@psau.edu.sa (O.A.); as.altamimi@psau.edu.sa (A.S.A.A.); m.alossaimi@psau.edu.sa (M.A.A.); 4 Department of Clinical Laboratory Sciences, College of Applied Medical Sciences, King Khalid University, Abha 62521, Saudi Arabia; irfancsmmu@gmail.com; 5 Smart-Health Initiative (SHI) and Red Sea Research Center (RSRC), Division of Biological and Environmental Sciences and Engineering (BESE), King Abdullah University of Science and Technology (KAUST), Thuwal 23955, Saudi Arabia; mariusz.jaremko@kaust.edu.sa; 6 Core Labs, King Abdullah University of Science and Technology (KAUST), Thuwal 23955, Saudi Arabia; abdelhamid.emwas@kaust.edu.sa; 7 Amity Institute of Pharmacy, Amity University, Gwalior 474005, Madhya Pradesh, India; tanweer0852@gmail.com

**Keywords:** skin cancer, nanoformulations, skin permeation, cutaneous squamous cell carcinoma, basal cell carcinoma

## Abstract

Cancer is a progressive disease of multi-factorial origin that has risen worldwide, probably due to changes in lifestyle, food intake, and environmental changes as some of the reasons. Skin cancer can be classified into melanomas from melanocytes and nonmelanoma skin cancer (NMSC) from the epidermally-derived cell. Together it constitutes about 95% of skin cancer. Basal cell carcinoma (BCC) and cutaneous squamous cell carcinoma (CSCC) are creditworthy of 99% of NMSC due to the limited accessibility of conventional formulations in skin cancer cells of having multiple obstacles in treatment reply to this therapeutic regime. Despite this, it often encounters erratic bioavailability and absorption to the target. Nanoparticles developed through nanotechnology platforms could be the better topical skin cancer therapy option. To improve the topical delivery, the nano-sized delivery system is appropriate as it fuses with the cutaneous layer and fluidized membrane; thus, the deeper penetration of therapeutics could be possible to reach the target spot. This review briefly outlooks the various nanoparticle preparations, i.e., liposomes, niosomes, ethosomes, transferosomes, transethosomes, nanoemulsions, and nanoparticles technologies tested into skin cancer and impede their progress tend to concentrate in the skin layers. Nanocarriers have proved that they can considerably boost medication bioavailability, lowering the frequency of dosage and reducing the toxicity associated with high doses of the medication.

## 1. Introduction

Cancer can be defined as uncontrolled cell division; it involves comparatively normal cells that divide without any control. Cancer has affected multicellular organisms for more than 200 million years; several shreds of evidence have been found in our ancestors of modern humans [1]. Edwin Smith Papyrus, written approximately 3000 B.C., was the first document describing disease and cancer. The earliest reference to soft tissue tumors, fatty tumors, and cancer of the rectum, stomach, uterus, and possibly skin is described in the Ebers Papyrus from 1500 BC [2]. The word cancer is derived from the Greek word “Karnikos” to describe the carcinoma tumors by Hippocrates (physician) in 460–370 BC [3]. Hippocrates described several superficial cancers in his book, “De Medicina”; Guy de Chauliac (1300–1368), who was a French surgeon, described skin disease and cancer in one chapter of “Chirurgia Manga” [2]. Laennec first defined the term melanoma in 1804 and basal cell carcinoma in 1827 by Jacob. UV-induced skin cancers were described in the 19th and 20th centuries. The oncogene and tumor suppressor genes were discovered in the 1970s as gene families related to the development of cancer [4]. Both intrinsic mutators (e.g., Gene instability) and extrinsic mutators (e.g., Oncogenic virus) are responsible for cancer. Genetic dysregulation promotes the activation of growth-promoting genes and the inhibition of tumor-suppressor genes. Other contributions to carcinogenesis come from genes that regulate apoptosis (programmed cell death), alleles that affect DNA repair, and genes that control cell growth and cell proliferation, and genomic integrity; instability of all these genes accelerates cancer development and also causes cancer in the young [5,6]. Pre-malignant lesions (e.g., Dysplasia and hyperplasia) are caused by genetic alteration or environmental factors (e.g., viral infection). Genetic alteration initiates monoclonal expansion, while viral infection initiates polyclonal expansion. The conversion of pre-malignant cells into malignant ones occurs due to the accumulation of genetic instability, which is responsible for the production of primary tumors. The cells of primary tumors at an early expansion stage are noninvasive and non-metastatic. Still, later multiple alterations or the instability of genes make the cells fully malignant, becoming invasive and metastatic [7].

Skin cancer, called cutaneous carcinoma, is a pre-eminent global health problem [8]. Skin cancer is increasing progressively worldwide. Skin cancer is classified into (1) Melanomas, which arise from melanocytes, and (2) nonmelanoma skin cancer from the epidermally-derived cells. They both consist of about 95% skin cancer. Basal cell carcinoma (BCC) and cutaneous squamous cell carcinoma (CSCC) are responsible for 99% of nonmelanoma skin cancer (NMSC), and 20% of all skin cancer is due to CSCC. Most skin cancer is held by or occurs due to BCC [9]. The most common cancer in the United States is skin cancer; one out of five Americans is affected by skin cancer. The fifth most common cancer is melanoma [10]. The World Health Organization (WHO) estimates that there will be 1.5 million new cases of skin cancer worldwide in the year 2020. In 2020, 325,000 new melanoma cases were detected globally, and 57,000 people died due to the disease. Surprisingly melanoma incidence rates in various nations and areas of the world vary substantially. In most parts of the world, men are more likely to develop melanoma than women (https://www.iarc.who.int/cancer-type/skin-cancer/, accessed on 28 April 2023). The American Society of Dermatology Association reported that one in five Americans would develop skin cancer once in their lifetime. Nearly 9500 persons in the United States are diagnosed with skin cancer daily, and more than 1 million Americans have melanoma. Out of 196,060 new cases of melanoma, 101,280 noninvasive (in situ, on the top layer of skin) and 94,780 invasive (into the deeper layer of skin) have been diagnosed in the U.S. in 2021. It has been reported that women have more incidence cases than men for both types of NMSC [11,12]. The highest incidence of skin cancer in Australia in 2018 was almost 300/1,000,000 people, and more than 5.4 million skin cancer cases were diagnosed in the United States in 2012 [12]. Recently in Europe, the incidence of malignant melanoma was reported to be up to 15 new cases/100,000 per year. This incidence varies depending upon the intensity of sun exposure, that is, 20 to 25 per 100,000 population in Central and Northern Europe. In Central Europe, SCC is almost 10 per 100,000 population [13]. The data from the Moscow Oncology Research Institute, Ministry of Health of the Russian Federation, for 2007–2017 reported that overall incidence (29.64/100,000) and mortality (0.70/100,000) rate of NMSC in Russia, respectively; 63% of women were diagnosed with NMSC in 733,723 patients [14]. Worldwide deaths from skin cancer in 2018 were reported to be almost 126,000. Since the last four decades, the number of new cases in the U.S. has risen two-fold [15]. The incidence rate of skin cancer in Europe has been found to increase to 40–50/100,000 per year in the following decades [16]. The incidence of NMSC in African and American women is 2%. A study from Filipino-Hawaiians in Hawaii reported that the incidence of BCC in Asian women and men was found to be 7.3 and 16.7 (per 100,000), and one study from China shows the incidence of BCC in Asian men 5.8 and women 6.4 (per 100,000), respectively [17]. Variation in melanoma incidence is based on race, with a lifetime risk of 2.6% for Caucasians, 0.1% for African Americans, and 0.58% for Hispanics [17,18]. The incidence of melanoma in the white-skinned population of North America, Northern Europe, Australia, and New Zealand has increased by 4–6% yearly [19]. According to data from the Japanese Journal of Clinical Oncology skin cancer incidence in countries in Asia (China, India, Japan, Republic of Korea, and Thailand) is reported to be lower than in countries in America (USA, Canada, Brazil) and Oceania (New Zealand and Australia) and Europe (Italy, Germany, France, and the UK) across all age groups [20]. Almost 90% of NMSC occurs due to exposure to U.V. radiation. BCC and SCC arise from the malignant transformation of keratinocytes and suppression of the cutaneous inflammatory response. The BCC is mainly distributed on sun-exposed sites that is 80% on the head and neck and 15% on the trunk; BCC is primarily subdivided into three types: (a) Superficial 30%, (b) Nodular 6% and (c) Morphea from 5% to 10% [21]. The appearance of melanoma is sporadic, and it develops in skin cells called melanocytes. Melanoma is the most lethal skin cancer because it can spread through the Lymphatic system [22].

The skin, the body’s biggest organ, and its outermost superficial protective layer, has a thickness of 16 mm and covers an area of about 1.72 m^2^. It acts as a line or barrier through which exogenous substances cannot enter the body. The epidermis, dermis, and hypodermis, which make up the entirety of human skin from the outside to the inside, comprise many layers of different tissues, cells, and appendages [23]. The three layers that make up the entire thickness of human skin are the epidermis (outer), dermis (middle), and hypodermis (inner). The stratum corneum (SC), the stratum lucidum, the stratum granulosum, the stratum spinosum, and the stratum germinatum are the different layers of the epidermis [24]. The SC and lucidum are non-vital layers made of lipids and corneocytes that act as a vital barrier against causing harm from radiation, stop the loss of body water, and block the entry of exogenous substances into the body [25]. Melanocytes are responsible for skin pigmentation, which later blocks harmful ultraviolet radiation [26]. Additionally, the epidermis regulates the selective permeation of substances (mainly SCs; less than 0.5 kDa) through the dermis. The dermis primarily regulates the epidermis’s body temperature, pressure, nutrition, and oxygen supply. The hypodermis, the skin’s deepest layer, serves as an insulator, protects the body against shock, and stores energy in the form of fat [25].

Due to a lack of melanin pigment, skin cancer is a prevalent cause of malignancy in the United States and is most common in Caucasian or fair-skinned individuals [27]. Melanoma and non-melanoma skin cancer are the two main categories [28]. Melanoma accounts for only 1% of all skin malignancies, but it is one of the most aggressive skin cancers, showing only a 15–20% five-year survival rate after advances in therapy [29,30]. Non-melanoma skin cancer (NMSC) caused by genetic and environmental factors represents approximately 95% of skin cancers. The *p53* gene is mutated due to UV radiation exposure, leading to loss of function [31]. One of the key contributors to the emergence of skin cancer is DNA damage. Ionizing radiation, biological therapy, the human papillomavirus (HPV), immunosuppression, and organ transplantation are additional risk factors [25,31]. Skin cancer can be treated using various conventional methods, such as surgery, cryotherapy, radiation, and photodynamic therapy (PDT), as well as more recent drug delivery technologies such as NPs, nanovesicles, nanoemulsions, and nanogels [32]. There are many complications associated with conventional therapy, such as toxicity, inflammation, scarring, and poor patient compliance, which can be mitigated using novel nanotherapies [25]. As far as current knowledge, there are not commercially available nanosystems for the topical delivery of bioactive molecules for skin cancer. Even though most nanoparticles (NPs) applications are still in the preclinical phases, some research has made significant progress and shown promise, prompting the examination of these nanotechnology products in clinical trials. Nuccitelli et al. [33] aimed to evaluate Nano-Pulse stimulation paclitaxel to treat seborrheic keratosis. In another investigation, investigators Lang et al. [34] assessed the effectiveness of paclitaxel-loaded topical nanoparticles for treating cutaneous metastases from nonmelanoma cancer. Several reports have shown that nanodrug delivery systems such as liposomes, cubesomes, transferosomes, ethosomas, niosomes, spongosomes, etc., are used for the topical administration of drugs through intracellular, transapendagic, and transcellular routes [25,32,35,36]. Nanotechnology methods provide an opportunity to improve the treatment of many forms of cancer including skin cancer. This review attempts to collect and discuss important studies using these nanotechnological techniques for skin cancer.

## 2. Molecular Pathways

Skin cancer cells depend on cell-signaling pathways for their growth, nutrition, and development. However, it has been seen that any alteration or distribution in their mechanism would lead to the growth of cancer tissue formation [37]. PI3K/Akt/mTOR pathways are hubs amongst various signaling transduction pathways involved in numerous physiological functions linking nutrients, growth factors, and energy availability to protein and lipid synthesis, proliferation, cell growth, survival, apoptosis, angiogenesis, and tissue development [38]. P13K (phosphatidylinositol 3-kinase)-AKT-mTOR (mammalian target of rapamycin) detected in SCC is reported as abnormally activated and is studied as one of the most oncogenic/pre-cancerous pathways [39]. The mTOR/P13K/AKT is involved in the progression of many cancers type and has been reported to be activated in several cancer types, including skin cancer [40]. Cell survival and proliferation are being affected by P13K/AKT pathway. The activation of downstream kinase occurs due to stimulation of the P13K/AKT pathway and thus includes mTOR, p70 ribosomal protein S6 kinase 1 (p70S6K), and 4E-binding protein 1 (4E-BP1). mTOR phosphorylates 4E-BP1 and inhibits its activity, which results in the activation of cellular translation machinery by inhibition of eIF4E. Inhibition of 4E-BP1 inhibits Eif4E and promotes the activation of the translation machinery. mTOR can direct activate p70S6K1, which activates the downstream target, ribosomal protein S6, and leads to protein synthesis initiation. mTOR is activated by UVB (290–320 nm), which results in abnormal activation and development of SCC [41,42,43,44,45].

**Figure 1:**
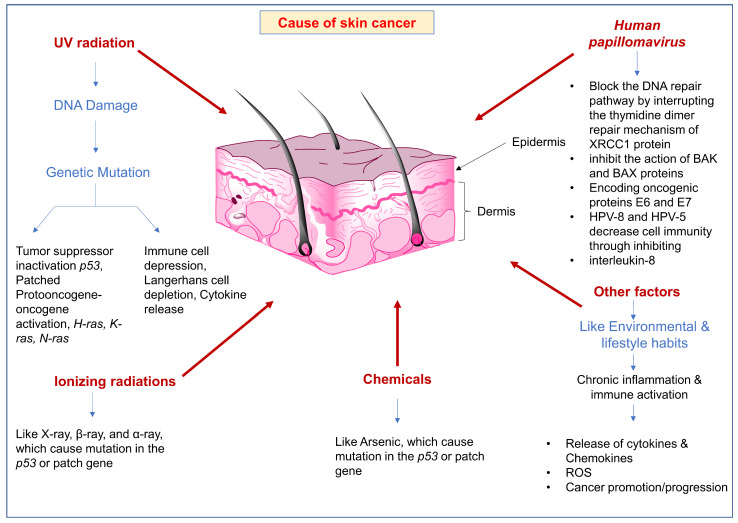
Different factors are involved in the progression of skin cancer.

The significant factors contributing to cancer include esophageal squamous cell carcinoma, lung cancer, and skin cancer, including over-expression and activation of V-akt thymoma viral oncogene homolog (AKT) and related signaling pathways. A rare genetic disease known as Xeroderma pigmentation is characterized by a defect in the ability of cells to repair the DNA photodamage that occurs due to exposure to U.V. radiation plays a crucial role in the development of skin cancer and increases the chance of developing skin cancer by nearly 1000-fold higher. Chronic exposure to U.V. radiation alters the activity of tumor suppressor gene p53 by inactivating via a broader range of mutations, further promoting carcinogenesis (Figure 1). Upon the sequencing of p53 genes of BSCC, many C to T mutations at di-pyrimidine sites and the tandem CC to TT mutations were reported, and these mutations occur due to UVB (290–320 nm) and UVC (100–280 nm). UV-radiation triggers Phosphatase and Tensin Homolog (PTEN) mutation on chromosome 6. Genetic mutation of PTEN, RAS oncogene (rat sarcoma virus), BRAF (a proto-oncogene), p53, CDKN2A (cyclin-dependent kinase inhibitor 2a), and altered activation of mitogen-activated protein kinase (MEK), T-LAK cell originated protein kinase (TOPK), 90 KkDa ribosomal S6 kinase (TSK). NMSC and AKT in melanoma induce the transduction of cancer cell signals and thus elevate skin carcinogenesis, the proliferation of cancer, migration, and cancer invasion [46,47,48,49,50,51]. The phosphoinositol-3-kinase (P13k)/AKT/mTOR and Raf/Erk signaling transduction modules are the best-studied Ras effector pathways. One of the essential oncogenic families of human cancer is Ras-genes, which include HRAS, KRAS, and NRAS and are responsible for activating mutations in almost 30% of solid tumors [51]. Ras-gene mutation influences or alters critical signaling pathways, such as Akt and mitogen-activated protein kinase (MAPK). The mutation of Ras can cause or result in the abnormal expression of the apoptosis-related protein. Ras-mutated cells are more prone to apoptosis and reactive oxygen species (ROS) attack than normal cells. Therefore, ROS activates the MAPK pathway, and this pathway involves in the regulation of cell survival and death [52]. It has been found that in almost 90% of melanomas, the MAPK pathway is hyper-activated, nearly 50% of all patients show a mutation in the kinase BRAF practically 28% of all patients bear mutations in the MAPK-pathway up-stream regulator NRAS. Excessive use of MAPK inhibitors in melanoma leads to the development of resistance, resulting in the vast complexity of MAPK signaling in a multicellular organism [53]. It has been found that the most prominent factor responsible for the development of skin cancer is U.V. radiation, which can take part in any step of carcinogenesis. All types of U.V. radiation from the sunlight can be divided into UVA (320–400 nm), UVB (280–320 nm), and UVC (100–280 nm); as the U.V. spectrum progresses from UVC to UVA radiation, the electromagnetic wavelength increases while the frequency decreases [53,54,55]. Figure 1 depicts the different factors involved in skin cancer development.

## 3. Nanoformulations-Based Delivery of Various Therapeutic Agents for Skin Cancer Rationale for Using Nanocarriers

There are multiple types of therapies available for treating skin cancer in both types: melanoma skin cancer and nonmelanoma skin cancer, including chemotherapy, radiation therapy, radiopharmaceutical therapy, hormonal therapy, active surveillance, immunotherapy, surgery, and gene therapy [55]. In recent decades, several drug resistances have been reported in the case of chemotherapy in in vitro and in vivo models, with many advantages.

The molecular resistance in skin cancer can alter the enzymatic system, dysregulation of apoptosis, faulty drug transport system, and changes in the apoptotic pathway [56]. For over five decades, approaches have been made for intralesional chemotherapy for melanomas skin cancer using drugs such as 5-fluorouracil, methotrexate, bleomycin, etc. The major drawback of intralesional chemotherapy includes a lack of large-good design trial, relatively small numbers of patients treated, off-label use of agents, and lack of therapeutic guidelines [57]. Another approach introduced at the beginning of the 20th century for topical treatment is Photodynamic Therapy (PDT) to kill skin cancer cells of both types (nodular BCC and SCC), which shows no drug resistance, high effectiveness, and is easy to use, but it causes prolonged phototoxicity. Additionally, the present PDT technology for treating BCC remains subordinate to Mohs surgery and surgical excision, and radiation therapy may develop radio chondritis in the patients. Treatment of cancer using multiple modalities such as electrosurgery, cryosurgery, Mohs chemosurgery, excision, and suture closure surgery are flexible options for the treatment of skin cancer, and these include wide advantages along with several demerits such as in the case of electrosurgery and cryosurgery, postoperative development of open wound and are postoperative bleeding. Excision and suture closure surgery is stressful and too long. In Mohs’ surgery, a 1% to 3% recurrence rate may develop, and some cancer cells can leave behind [58,59,60]. The most preferred route for the administration of drugs is the oral route, but many drugs (BCS class II and IV) show low aqueous solubility and permeability. The bioavailability of these drugs is very low due to variations in pH, from acidic pH 1 in the stomach to basic pH 8 in the intestine, hindrance in drug permeation due to intestinal mucosa, enzymatic degradation, and many other factors limit the clinical application [61].

**Figure 2:**
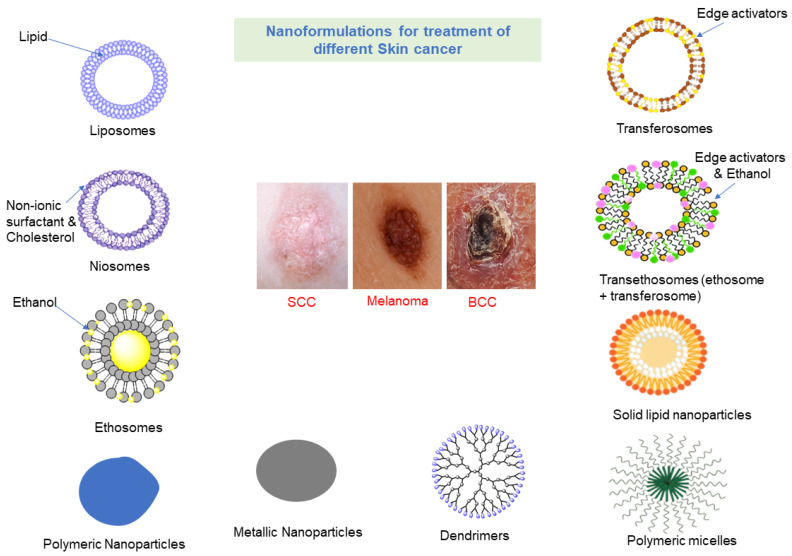
Different nanoparticle formulations were developed for the treatment of skin cancer.

Nanotechnology-based medical formulations (Figure 2) have attracted growing interest and led to the emergence of novel/nanoformulations of medicines [62]. Nanocarrier-based delivery systems have distinct chemical and physical properties, making them a potential candidate to be used as a synthetic platform for imaging probes in detecting and monitoring cancer and enhancing cancerous patient hope. A versatile nanostructured (Table 1) material, i.e., nanoemulsions, liposomes, quantum dots, ceramic-based carriers, polymeric nanoparticles, micelles, nano-shells, metal nanoparticles (gold, silver, iron oxide, titanium), dendrimers, carbon-nanotube, is the latest application in different anticancer treatments [63]. Through these delivery systems, unwanted exposure of normal cells is protected, resulting in sustained-release action to target cells [64]. The anticancer effect of transferrin-mediated solid lipid nanoparticles holding curcumin strengthens breast cancer [65]. RGD peptide-paclitaxel incorporated PEGylated PLGA-based nanoparticle enhances tumor endothelium targeting and intensifies the paclitaxel’s antitumor effect [66]. Conventional formulations of 5-fluorouracil (whose plasma half-life is about 10–20 min, and clinical use is limited due to stomatitis and myelotoxicity) show poor skin permeation. In contrast, the topical delivery of the transethosomal gel of 5-fluorouracil predicted excellent (83.67%) ex-vivo skin permeation. The entrapment efficiency and particle of optimized formulation were reported to be 92.6% and size of 57 nm [67,68]. Fisetin-loaded binary ethosomes enhance the anticancer effect and reduce the concentration of TNFα and IL-1a (cytokines) in UVB-exposed mice. Entrapment efficiency (89.23 ± 2.13%) and flux (1.01 ± 0.03 µg/cm^2^/h) rate across skin were reported [69,70]. Myricetin has limited water solubility and bioavailability. Lower particle size (291.11 nm) and higher encapsulation effectiveness percent (93%) were found for myricetin-loaded nanophytosomes, which improved their physicochemical stability and bioavailability [71]. Vincristine has nonspecific biodistribution and has serious side effects. Magnetic nanoparticles of vincristine boost the cytotoxic effect when P53 gene expression levels rise. P21, Caspase-9, and AKT-1 were down, as well as a drop in cancer cells. The nanocarrier showed a controlled release. The half maximum inhibitory concentration (IC_50_) values were calculated higher than 5 mg/mL and ten times the lethal concentration of the unbound drug [72]. Topical nano-delivery of vismodegib enhances the in vitro performance and skin penetration while reducing the in vivo toxicity [73]. Compared to the standard vismodegib formulation, the encapsulation of vismodegib into ultra-deformable liposomes boosted skin penetration (*p* < 0.0005). It inhibited the BCC’s hedgehog signaling pathway roughly seven times [74]. Compared to oral vismodegib, the optimized formulation of vismodegib loaded on ethosomal gel revealed a localized action and hence significant anti-tumor efficacy and a significant (*p* < 0.05) decrease in papilloma quantity [75]. Different approaches have been made to sort out this issue, such as the progression of the drug delivery system, such as liposomes, ethosomes, nanoemulsions, nanoparticles, and transethosomes [76,77,78].

## 4. Vesicular Nanoformulations for Skin Cancer

There has been much interest in developing novel drug delivery systems (NDDS) in recent decades. In an ideal world, the NDDS would satisfy two criteria: It should provide the medicine at a rate set by the body’s needs during treatment. Secondly, the active object must then be sent to the action location. The vesicular nanocarrier system is one of the most preferred delivery systems and is helpful in immunology, membrane biology, diagnostics, and, most recently, genetic engineering [93,94]. Various types of vesicular systems, extensively explored as nanoformulations in skin cancer treatment, are discussed here.

### 4.1. Liposomes

**Figure 3:**
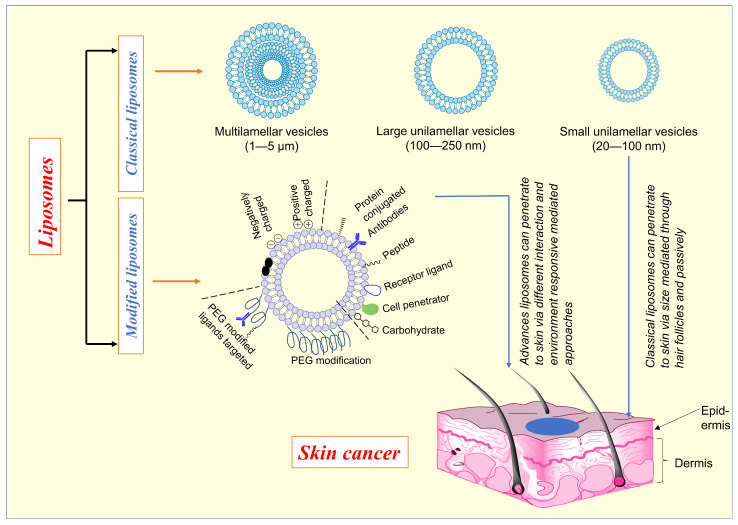
Depicts the various types of liposomes and the mechanism of penetration in skin cancer.

Dr. Alec D Bangham FRS, a British hematologist, initially described liposomes in 1961 (published 1964) at the Babraham Institute in Cambridge. Liposome comes from two Greek words: ‘Lipos’, which means fat, and ‘Soma’, which means body [95]. Liposomes are colloidal or microparticulate carriers with a 0.05–5.0 µm diameter. Drugs with a wide range of lipophilicity can be contained in liposomes within the phospholipid bilayer, the entrapped aqueous volume, or at the bilayer interface [96]. Single or more lipid bilayers are generated via hydrophilic and hydrophobic interactions with the aqueous phase in liposomes. In addition, Phosphatidylcholine (PC) and Dipalmitoyl PC can be utilized to make liposomes [97,98]. Various liposome types are available and depicted in Figure 3 for different diseases, including skin cancer. The formation of bilayers depends upon the ingredients utilized in the formulation. The types of each vesicle are unique in their way [99,100].

Liposomes are a well-established technology platform with many clinical applications [99]. Upon topical application, liposome uptake by the stratum corneum is most significant for positively charged liposomes, least for negatively charged liposomes, and least for neutral liposomes, implying that the electrostatic adsorption is the initial interaction between the corneal surface and liposomes [101]. The liposomal formulation might be enhanced pharmacokinetics, lower organ toxicity, and the potential to increase tumor absorption [102,103,104]. Various types of liposomes (Figure 3) were studied to improve skin penetration, controlled drug released, accumulation of drugs at specific sites, etc., to enhance the cytotoxic activity against skin cancer.

Endothelial growth factor receptors (EGFRs) are overexpressed in SCC, and responsible for poor prognosis and malignancy. EGFRs can be used to target the treatment of cancer. Petrilli et al. [101] have prepared the liposomal system loaded with 5-FU and co-administered with anti-EGFR (cetuximab) antibodies for targeting EGFRs. The uptake of EGFR-targeted 5-FU loaded liposomes showed about 3.5 times more uptake than control non-EGFR targeted liposomes. The antitumor efficiency of EGFRs-targeted showed more than 60% higher than the control liposomes when administrated subcutaneously. Another study by Singh [105] developed a liposomal system for dual targeting. Prepared liposomes meant for the targeting of AKT and COX-2. Liposomes were prepared and loaded with the combination of Dox and celecoxib and evaluated the cytotoxic efficacy in skin cancer and found that cancer cell viability inhibition was more than 99% at low concentrations, compared to alone. Multiple targeting might be a better treatment option for skin cancer treatment. The charge of tumor cells is more negative than normal cells [106,107]. One of the approaches to target skin cancer cells to cancer surface charged derived nanoformulations to enhance the cytotoxic effect of drug carriers. Jose et al. [108] used the cell surface charge to improve the cellular uptake of liposomes by using anti-STAT3 siRNA DOTAP-based cationic liposomes of curcumin. The cell viability of the co-delivery of curcumin and STAT3 siRNA using cationic liposomes in B16F10 mouse melanoma cells was inhibited considerably compared to either liposomal curcumin or STAT3 siRNA alone. The cationic liposomes are efficient drug delivery for the delivery of drugs or siRNA to the cancer cells for the treatment of skin cancer. The pH of the tumor microenvironment is acidic in comparison to the normal tissue environment [109,110]. pH-triggered drug delivery for delivery of drug to the tumor cells might be another approach. Lee and Nu [111] developed the pH-gradient anthocyanin-loaded liposomes for enhanced skin and improved cellular uptake. The antioxidant effect and skin permeation of formulation have significantly increased in this approach. The concentration of ROS was decreased by this approach, which later enhanced the cytotoxic effect in skin cancer. Under oxidative stress conditions, ROS production is significantly increased, leading to the oxidation of cellular proteins, lipids, and ultimately DNA, resulting in lethal lesions in cells that aid cancer development [109]. Another approach to enhance the penetration of drugs through the skin barrier and cellular uptake in skin cancer is deformable liposomes. Liposomes with edge activators increase skin permeability by reducing the stiffness of the bilayer structure and causing it to deform [112]. Marwah et al. [113] developed the deformable liposomal formulation using the tween 80 (edge-activator) and loaded with epigallocatechin gallatein (has antineoplastic properties) and evaluated the cellular uptake and cytotoxicity on HDFa and HaCat cells. The cell viability inhibition of formulation was found to be significantly below 100 µM and high cellular uptake in cancer cells. In a similar study, El-Kayal et al. [114] developed permeation enhancer-containing vesicles loaded with epigallocatechin-3-gallate for skin cancer treatment. The vesicles showed good inhibitory action against the A431 cells and reduced tumor size in the mice model. In another study, Sharma et al. [115], developed the C-type lectin receptor targeted nanoliposomes conjugated with mannose for the cross-presentation of ovalbumin as a model antigen. According to the findings, nanoliposomes dramatically increased antigen intake and cross-presentation to elicit CD8+ cell-mediated cellular immunity.

Liposomes are important drug delivery carriers for treating cancers, including skin cancer. Due to the penetration of skin barriers, classical liposomes have limitations in skin cancer treatment. However, the gradual improvement in liposomes, such as deformation liposomes, surface-modified liposomes, dual-targeted liposomes, etc., has better skin layer penetration and cytotoxic effects.

### 4.2. Niosomes

Niosomes are microscopic lamellar structures ranging in size from 10 to 1000 nanometers. Surfactants are non-immunogenic, biodegradable, and biocompatible makeup niosomes [116]. The two main components employed in forming niosomes are cholesterol and nonionic surfactants. Surfactants are essential in creating niosomes, whereas cholesterol provides stiffness and appropriate shape. For the manufacture of niosomes, nonionic surfactants such as spans (20, 40, 60, 85 and 80), tweens (20, 40, 60 and 80), and Brij (30, 35, 52, 58, 72 and 76) are commonly utilized [117,118]. Niosomes have amphiphilic characteristics, allowing hydrophilic medications to be entrapped in the core cavity and hydrophobic pharmaceuticals to be entrapped in the nonpolar area of the bilayer [119]. The alkyl chain length affects the surfactant’s hydrophilic-lipophilic balance (HLB) value (the lower the HLB value, the lower the entrapment efficiency). The studies found the most efficient entrapment between tween 20 and span 60 [120]. L’Oréal was the first company (in the cosmetics sector) to develop and patent nonionic surfactant niosomes [121]. Nonionic surfactants are nonpolar and polar sections with high interfacial tension that commonly form bilayers when hydrated or upon hydration [122]. Ether Injection, Hand Shaking, and Reverse Phase Evaporation are the most common methods for making niosomes [123]. Deformable niosomes are a mixture of nonionic surfactants, ethanol, and water. These tiny vesicles can readily pass through the pores of the stratum corneum, causing an increase in penetration effectiveness [124,125]. Table 2 indicates a comparison between niosomes and liposomes [126].

Its key features are the increased permeability of niosomes through the SC and the ability to reach the desired site of action [127]. Both hydrophilic and lipophilic drugs can be added to vesicular systems containing nonionic surfactants without causing toxicity. Resveratrol has poor bioavailability, low water solubility, chemical instability, and restricted skin permeability. The niosomal hydrogel system of resveratrol increases the permeation and deposition in the skin, enhancing the therapeutic action of resveratrol [128]. Chermahini et al. [129] developed the 5-FU-loaded for skin cancer, and results suggested that niosome-encapsulated fluorouracil showed significantly higher anticancer activity of the niosomal formulation when compared to other treatments with niosomes, which might be due to the enhanced skin permeability of niosomes. In a similar study by Paolino et al. [130], the study showed that 5-FU loaded alpha,omega-hexadecyl-bis-(1-aza-18-crown-6) and span 80 containing the niosomal system has several folds more cytotoxic effects (in SKEML-28-cells) than the control; it might be due to the enhanced permeation enhancement of niosomes. Topical use of niosomes may increase the residence time of the drug in the stratum corneum and epidermis while reducing the systemic absorption of the drug [129]. Pawar et al. [131] developed the *N*-lauryl glucosamine conjugated Doxorubicin (Dox)-loaded nanoniosomes to target specific drug delivery for the treatment of cancer. The results suggested that conjugated nanoniosomes have more cytotoxicity efficacy against cancer and are less toxic against normal cells than non-conjugated systems. Shah et al. [132] prepared a niosomal gel containing the antioxidant Gamma oryzanol. The permeation through the skin was enhanced and might be an improved option for skin cancer treatment.

### 4.3. Transferosomes

Gregor Cevc coined the term “transferosome” in 1991. The name “Transfero” comes from the Latin word “transfero,” which means “to carry across,” and the Greek word “soma”, which means “body” [133]. Transferosome comprises one inner aqueous compartment and is enclosed by a lipid bilayer with an edge activator. Transferosome (a vesicle) has both self-regulating and self-optimizing properties. Transferosomes are elastic and can deform, squeeze and cross the skin’s stratum corneum. Edge activators fluidize or solubilize the skin’s lipids, enhancing skin permeation [134]. Transferosomes have higher entrapment efficiency, flux, and deposition when compared to liposomes and niosomes [135]. Various types of ingredients used in the formulation of transferosomes and their role are given in Table 3.

Transferosomes, also known as deformable vesicles, have increased drug delivery to the skin. Enhanced amounts of both small and large therapeutic agents are delivered into and through the skin using transferosomes [82]. Jangdey et al. [136] developed ultra-flexible lipid vesicles such as an apigenin-loaded transfersomal system for the enhanced skin delivery of skin cancer treatments. The result suggested that the permeation of the drug through the skin via transferosomal formulation was significantly higher (*p* < 0.05) than that of the marketed product. The permeation of cytotoxic moieties in the tranferosomal system can improve skin cancer treatment. Another investigation by Sivarajakumar et al. [137] prepared the paclitaxel-loaded transfersomal vesicular systems to enhance the delivery of drugs at the site of skin cancer cells by improving the permeation through the skin. The results suggested that optimized transfersomes had a flux of 6.68 ± 0.46 and a percent drug retention of (0.79 ± 0.05) through the skin of mice. In another investigation, Jangdey et al. [138] developed the concanavalin-A conjugated nanotransfersomal gel of apigenin for enhanced targeted delivery of UV-induced malignant melanoma, which binds directly to the melanocytes gel layer in UVB-induced skin carcinoma. According to the findings, the cytotoxicity of concanavalin-A conjugated nanotransfersomal gel against A375 in a concentration range of 0.4–2.0 mg/mL, but less toxicity toward HaCaT cells. According to these investigations, transferosomes can increase cytotoxic efficacy by enhancing drug penetration at the site of action and cellular uptake.

### 4.4. Ethosomes

Ethanolic liposomes are also called ethosomes. Ethosomes are noninvasive delivery vehicles that allow medications to be delivered deep into the skin’s layers and/or the circulatory system. These soft, pliable vesicles are designed to distribute active substances more effectively [139]. Ethosomes comprise phospholipids, ethanol (higher concentration), and water. Ethosomes range in size from tens of nanometers (nm) to microns (µ) and permeate the skin layers more quickly, and transdermal flux is substantially higher [140]. Touitou named the term ethosomes, and high concentration of ethanol (20–50%) is the main reason for better skin permeation. Ethosomal formulation can disrupt the lipid bilayer structure of the skin and penetrate the stratum corneum (which possesses a very compact structure) [141]. Ethosomes enhance the lipid fluidity of the cell membrane and reduce the density of multi-layered lipids of the cell membrane, where it binds to skin lipids and release drugs into the deeper layers of the skin [142].

Ethosomes may be divided into the classical and binary ethosomes. Classical ethosomes show improved skin penetration and stability profiles compared to classic liposomes. The molecular weights of pharmaceuticals trapped in classical ethosomes have increased, ranging from 130.077 Da to 24 kDa [143]. Binary ethosomes were first introduced by Zhou. These were created by mixing another alcohol with the traditional ethosomes. Propylene glycol (PG) and isopropyl alcohol (IPA) are the most often employed alcohols in binary ethosomes [144,145].

**Figure 4:**
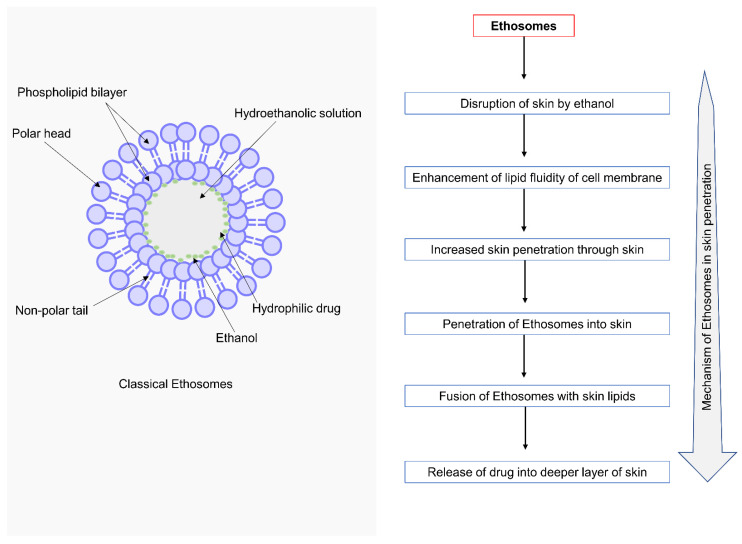
Schematic representation of the classical ethosomes and mechanism of action ethosomes.

Several studies have demonstrated the superiority of an ethosomal carrier over other nanocarriers, implying that it has a significant impact on drug delivery systems. Table 4 shows additives for the formulation of ethosomes and their functions [146]. The systematic presentation of the mechanism of action of ethosomes is given in Figure 4. Ethosomes, when applied topically, disrupt the skin, increase the lipid fluidity of the skin, and promote penetration across the skin. Later they fused with the skin and released the drug gradually [147].

An investigation by Gamal et al. [83] prepared the sonidegib-loaded ethosomes and later incorporated them in the gel to effectively treat skin cancer. Ethosomal formulation of sonidegib depicts a mean size of (199.53 ± 4.51 nm), steady-state flux (5.58 ± 0.08 µg/cm^2^/h), and entrapment efficiency of (87.5 ± 2.5); this exhibits that ethosomes possess higher entrapment efficiency and the other physiochemical properties are acceptable. The antitumor efficacy of ethosomal formulation showed significantly higher relative anti-tumor activity and 3.18 times bioavailability than the oral sonidegib drug. Another study by Peram et al. [147,148] prepared the curcumin-loaded ethosomes. The optimized curcumin-loaded ethosomes significantly reduced (*p* < 0.05) the cell viability of A375 cells compared to free curcumin. It could be attributed to the persistent release of curcumin from ethosomes, resulting in continued drug exposure to tumor cells and more significant anticancer activity. Mousa et al. [149] prepared the metformin-loaded ethosomes for skin cancer treatment. Results showed the high permeation efficiency of ethosomes through the skin and higher antitumor efficiency than the pure drug. The small-sized ethosomes penetrate deeper into the skin layer, later causing improved antitumor efficacy.

The formulations are a few examples of ethosomes, which have small-sized, better skin layer penetration and significantly high anticancer activity. The ethosomal formulation gives hope for a better treatment strategy for skin cancer treatment.

### 4.5. Transethosomes

Transethosomes are vesicles with an irregular shape, and the size lies between 40 nm and 200 nm depending upon the size of the drug. Transethosomes are a type of UDV (ultra-deformable vesicle) and a novel lipid vesicle that is flexible and deformable. UDVs were developed at the beginning of the 1990s and could deliver the drug into the epidermis or skin’s dermis and deep circulation [150]. Transethosomes (TELs) consist of phospholipids, ethanol, water, and edge activators (surfactants) or permeation enhancers (e.g., oleic acid). The edge activators (surfactants) are single-chain surfactants that provide flexibility by destabilizing the vesicle’s lipid bilayer, which in turn reduces interfacial tension and augments its structure deformability [148]. When substantial levels of ethanol (about 30%) are combined with edge activators, a synergy is created that allows transethosomes to enter and disperse deep into the epidermis. Lipid bilayer rearrangement and ethanol aid in enhancing the solubility of lipophilic medicines and disturbing the SC. As a result, transethosome can carry medications deep into the dermal layers or even the systemic circulation [151]. Moolakkadath et al. [67] developed a fisetin-loaded transethosome delivery system for nonmelanoma skin cancer. They optimized the formulation using the Box–Behnken design and found that the optimized formulation had nano-range vesicle size (74.21 ± 2.65 nm) possessing good entrapment efficiency (68.31 ± 1.48%) and good flux (4.13 ± 0.17 mg/cm^2^/h) for fisetin dermal delivery. These formulations showed high penetration through the skin, providing a better treatment strategy for skin cancer treatment. Abdulbaqi et al. [151] concluded that the permeation of transethosomal gel has superior permeation properties than non-transethosomal gel. Another study by Abdulbaqi et al. [151] developed colchicine-loaded transethosomes to enhance skin penetration. The findings found that transethosomal gel has better stability at refrigerated conditions (4 °C ± 2 °C) and high skin permeation efficiency. The study concluded that transethosomal gels are potent carriers for the transdermal delivery of colchicine.

Transethosomal drug delivery is one of the important drug delivery systems for the delivery of drugs or other active moieties from the skin due to their better skin Lipid Nanoparticles (LNPs).

Solid Lipid Nanoparticles (SLNs) and Nanostructured Lipid Carriers (NLC) are lipid nanoparticles that are very stable; well tolerated and protect medications from degradation while ensuring a consistent release over time [94,152].

### 4.6. Solid Lipid Nanoparticles (SLNs)

SLNs are a colloidal system with sizes ranging from 50 to 1000 nm. A high-pressure homogenization process combines biodegradable and biocompatible solid lipids, emulsifiers, and water. Triglycerides, glycerides, and other lipids commonly employ waxes and fatty acids [153]. SLNs form a monolayer on the skin, creating an occlusive effect that increases water retention inside the skin [154]. Tupal et al. [153] prepared and optimized the formulations of Dox-loaded solid lipid nanoparticles and optimized showed a mean particle size of 92 nm and entrapment efficiency of 86% following a 40-day study period, both the low and high doses of Dox-loaded SLNs were found to produce significantly better results than free Dox formulation (*p* < 0.05). In terms of tumor volume and weight assessments, there was no statistical difference between the low and high doses of Dox-SLNs (*p* > 0.05) [84]. Studies by Kaur et al. [155,156] prepared the curcumin-loaded SLNs for evaluating the anticancer activity, stability, permeation, and pharmacokinetics parameters. The results suggested that SLNs have high permeation, stability, bioavailability, and anti-cancer effects. The incorporation of SLNs in gels or patches can improve the treatment of skin diseases, including skin cancer. In this prospect, a study was performed by Sabir et al. [157]. They tested this approach, prepared the curcumin-loaded solid lipid nanoparticles, and incorporated them into the patches for transdermal delivery. The prepared particles showed a mean particle size of 170 ± 2 nm with an entrapment efficiency of 90 ± 3.5% (*w*/*w*). The permeation efficiency of SLNs incorporated patches has about 6.5 folds permeation than the non-patches SLNs. In another study by Gonçalves et al., which prepared the cutaneous SLNs and loaded multiple natural compounds such as naringenin, nordihydroguaiaretic acid (NDGA), and kaempferol and found that the SLNs had a mean particle size of 200 nm with high drug entrapment efficiency. Formulations were evaluated against human keratinocytes (HaCaT) for anti-cancer activity. The formulation reduced the ROS, produced due the oxidative stress and provides significant anticancer activity. In another study by Banerjee et al. [158] Tyr-3-octreotide modified SLNs loaded with Ptx were prepared for specific targeting for the highly expressed somatostatin receptors present on the melanoma cells to enhance the treatment of the same. The results of the study showed that Tyr-3-octreotide exhibits remarkable anti-melanoma activities without any observable toxicity. Several pieces of research showed that SLNs permeate through the skin, penetrate the deeper layers, and reach target sites. These formulations might be effective carriers for skin cancer treatment.

### 4.7. Nanostructured Lipid Carriers (NLCs)

NLCs are made up of solid and liquid lipids and have a crystalline structure that is not perfect. The lipid is either encased in a solid lipid matrix or is found on the surfactant layer [159]. The solid lipid component imparts properties for controlled drug release, whereas the liquid phase with lower water content provides substantial drug loading [85]. Furthermore, enhanced drug loading in NLCs is supported by increased distances between the fatty acid chains and the unstructured crystal [160]. They are significantly more appropriate for medication formulation than SLNs. NLCs are simple to make and are produced using pressure homogenization, nanoemulsion, or aqueous dispersion procedures. [63,161].

Moradi et al. [162] developed the NLCs containing Tretinoin as an active moiety to improve skin uptake and reduce the side effects. The findings demonstrated that a prolonged release profile maintains tretinoin penetration and absorption while promoting skin tolerability. Iqbal et al. [163] also prepared the silymarin-loaded NLCs and later incorporated them in the gel to treat skin cancer against the B16 melanoma cell line. According to the results, the group treated with the silymarin-NLC gel appeared to have significantly higher levels of superoxide dismutase, catalase, and glutathione and significantly lower levels of IL-1α and TNF-α. The antitumor effect of silymarin-NLC gel was also evaluated, and the results showed a significant (*p* < 0.05) reduction in tumors. Another study by Gundogdu et al. [164] developed the Imatinib (tyrosine kinase enzyme inhibitor)-loaded NLCs for cancer treatment. Besides the physiochemical properties of the formulation, the anticancer effect was evaluated against the CRL-1739 cell line. The formulation showed 23.61 µM of IC_50_ and induction of apoptosis in the cancer cells. The cytotoxicity efficiency of the formulation was significantly high in cells.

## 5. Polymeric Micelles and Nanoparticles (NPs)

### 5.1. Polymeric Micelles

Self-assembling polymer chains can produce micellar-like particles with a hydrophobic core and a hydrophilic exterior. The inner core can integrate poorly soluble medicines for improved bioavailability, while the outer hydrophilic corona keeps the core stable in aqueous environments [63,161]. Polymeric micelles typically range in size from 10 to 80 nm. Micelles can be functionalized with ligands to improve efficacy and specificity (for example, antibodies, peptides, aptamers, carbohydrates, and small molecules) or block copolymers that release the medication in response to chemical and/or physical stimuli [165]. Quiñones et al. [166] the celecoxib-containing polymeric micelles were prepared to prevent and treat inflammation and cancer, mainly focused on skin cancer. The findings suggested that the efficacy of inflammation and UVB-induced skin cancer treatment was increased and minimized the toxicity. The actively targeted micelles have been prepared to improve the efficacy of the drug delivery system and deliver the drug/active moieties to the target site. The fundamental goal of active targeting is to enhance drug delivery to a target region through specific interactions, such as antibody and antigen binding, or locally applied cues, such as heating and sonication [165]. In this prospect, several studies have been performed. Lapteva et al. [167] developed the biodegradable and biocompatible methoxy-poly (ethylene glycol)-poly(hexyl substituted lactides) diblock copolymer to produce the ciclosporin A-loaded micelles to test their potential to deliver the drug selectively into the skin without concurrent transdermal permeation. The result suggested that skin penetration was high in these structures, indicating that intergroup penetration is the preferred transport channel for enhanced dermal delivery of ciclosporin A. These studies proved that polymeric micelles are stable and nano-sized drug delivery carriers with high penetration do so efficiently, passively and actively to the skin, which might be a valuable tool for treating cancer.

### 5.2. Polymeric NPs

Polymeric NPs are one of the extensively studied NPs for the delivery of different types of drugs, phytoconstituents, natural products, etc., for the treatment and diagnosis of various diseased conditions [59,168,169,170,171]. Polymeric nanoparticles have been shown to permeate the skin through the follicular system [172]. Chitosan-based NPs have been extensively studied for cutaneous medication delivery among natural polymers. Chitosan is a biodegradable and cationic N-deacetylated derivative of chitin. The positive charge allows the polymer to engage aggressively with the negatively charged skin surface, altering the barrier and allowing medications to be delivered [86]. In addition, the polymer is antioxidant, anti-inflammatory, and antimicrobial. These properties make it ideal for treating skin conditions [173]. Valizadeh et al. [174] developed the polymeric natural polymeric NPs of chitosan for skin and breast cancer. *Syzygium aromaticum* essential oil and eugenol were used as active ingredients and evaluated the antioxidant effects as cytotoxic treatment. The Ic_50_ of chitosan nanoparticles containing *Syzygium aromaticum* essential oil and eugenol against melanoma (A-375) cells were found to be at 73 and 79 μg mL^−1^, respectively. This cytotoxic effect was significantly more than the control. Similarly, in another study, Neelakandan et al. [175] developed chlorogenic acid–loaded chitosan NPs and evaluated their anticancer activity. The results suppressed the tumor significantly and maintained the level of oxidative stress when given topically compared to oral administration in mice.

Poly (ε-caprolactone), polylactic acid (PLA), and poly (lactide-co-glycolide) copolymer (PLGA) have been investigated for dermatological purposes among synthetic biodegradable polymers. In an imiquimod-induced psoriasis model in mice, Sun et al. colleagues found that curcumin-loaded PLGA (50 nm to 150 nm) had a better therapeutic effect than the curcumin hydrogel model [173]. Due to their small size, polymeric particles can form depots by accumulating within the hair follicle and on the skin surface [176]. A study by Das et al. [177] developed the apigenin-loaded-PLGA NPs for the treatment of UVB and Benzo(a)pyrene (BaP) induced skin tumors. The nanoparticles were evaluated for the mitochondrial transmembrane potential, ROS accumulation, cytochrome c, expressions of Apaf-1, bax, bcl-2, cyt c, cleaved caspase-9 and 3, etc., which are signs of the cytotoxic effect of treatment. The results showed clear signs of cytotoxicity in skin cancer cells. These NPs showed the potential to improve the combat against skin cancer, and therefore, have tremendous potential for use in the curative management of skin cancer. Targeted polymeric nanoparticles bind to specific overexpressed targets or antibody-antigen interactions, which might provide improved targeted drug delivery and reduce the side effects of a cytotoxic drug to adjacent normal healthy cells. Yaman et al. [178] prepared the trametinib-loaded PLGA NPs, and the surface was modified by coating T cell hybridoma, 19LF6 with anti-gp100/HLA-A2 T-cell receptor. According to the research, the NPs had a tumor retention rate more than double that of the non-specific membrane-coated and uncoated groups. These T-cell membrane-coated NPs are emerging as promising diagnostic carriers for melanoma-related imaging and therapeutic applications.

Several polymeric NPs such as silk fibroin [168,179], silk sericin [180], polycaprolactone [181], β-cyclodextrin [182], albumin [183], etc., are extensively studied for the treatment of several cancers, including skin cancer. Owing to their biostability, biocompatibility, ease of fabrication, etc., polymeric NPs are the most used NPs in skin cancer treatment. Therapeutic substances are sustained release, and there is also a possibility of drug entrapment. Cytotoxic drugs, siRNA, genes, proteins, enzymes, and other substances can be attached to polymeric NPs. The general efficacy of polymeric NPs makes them a promising delivery mechanism for various diseases and skin cancer.

### 5.3. Dendrimers

Dendrimers are synthetic polymer-based monodisperse NPs. A center core exists in each particle, which gives rise to symmetrically ordered repeating units, resulting in a layered architecture. The functional groups in repeating units grow at an exponential rate. Dendrimers are highly monodispersed, multivalent, and have a well-defined size [184]. Dendrimers have many surface functional groups and internal cavities due to their high degree of branching. Furthermore, dendrimer core-shell architecture includes lipophilic and hydrophilic molecules [185]. Dendrimers permeate the skin because of the following characteristics: particular surface charge, hydrodynamic size, molecular weight, generation size, composition, and concentration [186]. Polyamidoamines (PAMAM), poly (l-lysine) (PLL) scaffold dendrimers, polyesters (PGLSA-OH), and other dendrimers are extensively employed in drug delivery [187]. Low-generation PAMAM dendrimers (G0-G4) have hydrodynamic radii of less than 5 nm, which enables them to penetrate the lipid matrix (intercellular) [188]. Dendrimers have been utilized for effective dermal delivery of various drugs, for example, anticancer, antiviral, alpha-blocker, peptides, NSAIDs, and antimicrobial and antihypertensive drugs [189]. To treat skin cancer, Ybarra et al. [190] developed and evaluated the PAMAM 4.0 and 4.5 generations dendrimers complexed with vismodegib (Hedgehog signaling pathway inhibitor). The results of the cytotoxic assay against the HaCaT cell line were 3.21 µM (IC50) was significantly lower than the control. A similar preparation was developed by Thuy et al. [191] for incorporating paclitaxel and curcumin in dendrimeric micelles for treating melanoma. Results of the study demonstrated that the formulation increased the aqueous solubility and bioavailability, which are limiting factors for their therapeutic applications. The drug-loaded dendrimeric micelles suppressed melanoma cells and had antibacterial properties. These data demonstrate the dendrimeric formulations’ theragnostic potential in skin cancer treatment.

## 6. Nanofibers

Nanofibers may be produced from various materials, including polymers, carbon, and semiconductors [192]. The nanofiber diameter is less than 100 nm, has a controllable pore size, and has a high surface-to-volume ratio [193]. Nanofibers for topical medication administration have been made of various natural and synthetic polymers. Nanofiber are made from gelatin, fibronectin, collagen, hyaluronic acid, silk fibroin, chitosan, gelatin, hyaluronic acid, silk fibroin, chitosan PLA, PLGA, PGA, polycaprolactone, poly (vinyl pyrrolidone), poly (vinyl alcohol), polyurethane, polycarbonates produced from tyrosine, and so forth [194]. Modulating the drug-to-polymer ratio, fiber diameter, shape, porosity, or surface functionalization may be used to make electrospun fiber mats that steadily release medications. Nanofibers can deliver anticancer, antioxidant, antifungal, wound healing, and local anesthetic therapeutics in a controlled and sustained manner [195].

Several types of research were conducted to evaluate the anticancer potential against skin cancer and are still being investigated. In another study, Patel et al. [196] prepared 5-FU-loaded chitosan and PVA electrospun nanofibers for skin cancer treatment. The study indicated that the nanofibers reduced cell viability by more than 50% after 24 h, while cell numbers decreased by 10% after 48 h. A study by Rengifo et al. [197] developed the polyethylene oxides-chitosan nanofibers containing carboxymethyl-hexanoyl chitosan/dodecyl sulfate nanoparticles loaded with pyrazoline for the treatment of skin cancer. The findings were favorable for skin cancer treatment. The cytotoxicity against B16F10 melanoma was high, suggesting that a prepared carrier system may be a potential approach for treating skin cancer. A similar study was performed by Balan et al. [198], in which the team developed polymeric nanofibers incorporated with resveratrol and ferulic acid-loaded nanoparticles. The anticancer effect of drug-loaded nanofibers was investigated using A431 cells, which showed a 30% and 50% decrease in cell viability when treated with nanoparticles and nanofibrous scaffolds, respectively.

These nanofibers releasing drugs at the site of action sustainably might be suitable for treating skin cancers.

## 7. Metallic NPs

Gold, silver, and metallic oxides are commonly used to make metallic NPs. These particles have been widely employed in a variety of skincare products. Drugs are either integrated or bound to the surface of the core [192,199]. Neutral and positively charged silver nanoparticles (AgNPs) in an aqueous solution penetrated the human skin more when compared to an oil-in-water emulsion and an aqueous solution vehicle [200]. Metallic N.P.s have an occlusive impact on the SC barrier or fluidize the barrier to allow for greater skin penetration. The particles can squeeze through the pores due to their deformability or flexibility. Polymeric NPs, on the other hand, accumulate deep inside SC or use the trans follicular route to establish a depot within the skin [201,202]. Depending on the material surface properties, metallic NPs have been demonstrated to gather superficially or within the skin. Skin-penetrating proteins are used to make these technologies more functional [203]. Alhoqail et al. developed husk-like zinc oxide nanoparticles and evaluated the anticancer activity against A431 cells and other physiochemical properties. The results showed that formulation increased nuclear condensation and ROS generation, which led to cell death and nuclear apoptosis and might be a prominent drug delivery carrier for cancer treatment. Safwat at al. [204] developed the 5-FU gold nanoparticles cream showed a 2-fold greater penetration through mouse skin when compared to free 5-fluorouracil cream. In vivo, investigations in a mouse model with A431 skin cancer cells implanted in the subcutaneous region revealed that the GNP (gold nanoparticle) cream reduced tumor volume by 18.4 times, respectively, compared to the untreated control. These findings demonstrate that metallic nanoparticles are one of the prominent drug delivery carriers for the treatment of skin cancer.

A treatment option for skin cancer called photodynamic therapy (PDT) uses photosensitizing drugs and light to kill cancer cells. A promising method for enhancing the effectiveness of PDT is the use of nanoparticles [205]. When used in PDT, the NPs may contain photosensitizing agents, which enhance their delivery to the tumor site [206,207]. Furthermore, by selectively targeting cancer cells, they can reduce damage to healthy tissue while increasing the selectivity and efficacy of PDT [208,209]. Photosensitizing agents such as porphyrin or phthalocyanine are loaded or contained within the nanoparticles. These agents are normally activated when exposed to light of a particular wavelength [210]. The NPs can be modified with specific ligands or antibodies so that they can specifically recognize and bind to cancer cells. This targeting ability accelerates the accumulation of NPs in tumors while inhibiting their uptake by healthy tissues. The nanoparticles can be modified with specific ligands or antibodies so that they can specifically recognize and bind to cancer cells. This targeting ability enhances the accumulation of nanoparticles in tumors and reduces their uptake by healthy tissues [211]. Once delivered, NPs can be targeted actively (specific recognition of cancer cells) or passively (leakage through abnormal tumor vasculature) to reach the tumor site. This increases the concentration of the photosensitizer in the tumor and increases the efficacy of the therapy [212]. After allowing time to accumulate at the tumor site, the photosensitizer-loaded nanoparticles are exposed to appropriate wavelengths of light [213]. ROS, which can damage cancer cells, is produced when light activates photosensitizers. Reactive oxygen species produced by photosensitizers that have been activated trigger a series of cellular processes that result in cell death [214,215]. ROS have the ability to disrupt cell membranes, cause oxidative damage to cancer cell components, and trigger apoptosis (programmed cell death) [109].

In this prospect, Reis et al. [216] prepared the NPs PDT loaded with dacarbazine and zinc phthalocyanine and evaluated the photodynamic efficacy against the MV3 melanoma cells. The in vitro results suggest that the developed PDT has significant cytotoxicity against the melanoma cells but no cytotoxicity in normal counterparts. Furthermore, in vivo results showed that drug loading affects the biodistribution of NPs. The low accumulation of NPs in the stomach, heart, brain and kidney suggested that the general side effects of dacarbazine could be reduced. In another study, Li et al. [217] prepared the polyglycerol-coated iron oxide NPs loaded with chlorin e6 (photosensitizer) and conjugated with Dox to enhance PDT for the treatment of melanoma. According to the findings, Dox-conjugated PDT enhances cellular uptake and exerts photocytotoxicity, as demonstrated by increased reactive oxygen species production, decreased viability, DNA damage and stimulation of tumor cell immunogenicity.

It is important to remember that although PDT nanoparticles have promise, research and development may still be ongoing for their clinical applications and formulations. To learn about the latest discoveries and treatment options for skin cancer, it is always best to speak with medical experts or researchers who specialize in this area.

## 8. Nanogels

Nanogels are three-dimensional polymeric cross-linked porous hydrogels with diameters ranging from 20 to 200 nm with gel and nanoparticle characteristics. Nanogel is a viable contender as a targeted drug delivery method in treating skin cancer due to its high entrapment efficiency, thermodynamic stability, solubilization potential, and swelling potential. To obtain the controlled release of drugs and other bioactive compounds, nano-gels are synthetically or architecturally changed to respond to internal or external stimuli such as radiation, ultrasound, enzymes, magnetic, pH, temperature, and oxidation-reduction [218]. Following interaction with SC, such nanogels can undergo a physical transition for greater dermal penetration and payload release in response to ionic strength, temperature, or the skin’s pH gradient [87]. For example, pH-responsive and biodegradable chitosan or PLGA-chitosan nanogels have been proven to release 5-FU in reaction to the tumor’s acidic environment to cure melanoma [219]. PEG-functionalized NPs or nanogels have also been shown to have better skin penetration. PEG can interact with keratin and solvate it, allowing the lipid content to be extracted and SC to be disrupted [220]. Dendrimers made of dendritic polyglycerol (dPG) were employed to create hydrophilic and thermo-responsive three-dimensional cross-linked nanogels that let small and big molecules penetrate SC and concentrate in hair follicles [221]. Badalkhani et al. [222] formulated γ-oryzanol-loaded NLCs and nanosized UV filters TiO_2_ to evaluate the synergistic potential for skin protection against diseases such as cancer. Studies have shown that nanogel loaded with NLCs and nano UV filters had excellent long-term storage stability and photoprotection capabilities, which might be useful for protection against UV-generated skin cancer. Several studies’ results as discussed here show the enhanced potential of nanogels against skin cancer, making the nanogels suitable candidates for the treatment and prevention of skin cancers.

## 9. Nanoemulsions (NEs)

The proportions of the droplet are a critical framework that distinguishes a NEs (d 200 nm) from a regular emulsion (d > 200 nm). As a result, traditional emulsions have a proclivity for degrading over time [223,224,225]. NEs are nanoscale thermodynamically stable dispersions of water in oil (*w*/o) or oil in water (o/*w*), stabilized by a surfactant interfacial coating [224,225,226]. Due to their tiny particle size, NEs are substantially more resistant to gravity separation and aggregation than ordinary emulsions. Microemulsion particles can be spherical or non-spherical, whereas nanoemulsion particles are generally spherical [225,226,227]. High-energy (e.g., high-pressure homogenization) or low-energy (depending on the physicochemical features of components) emulsification procedures are the most common methods for creating NEs [226]. Its vast surface area allows it to make close occlusive contact with the stratum corneum, which aids in perspiration and transports medications deep into the skin’s surface. The presence of oil and surfactants enhances the penetration of NEs through the skin [227].

Martínez-Razo et al. [228] developed the NEs to evaluate their potency against skin cancer treatment. In this study, norcantharidin-loaded oil-in-water NEs were prepared and evaluated the cytotoxic potential against B16F1 cells, and IC_50_ was found to be 1.026 ± 0.370 mg/L, significantly lower than the control. In another study, Ranjbar et al. [229] prepared the *Cuminum cyminum* essential oil containing NEs and evaluated the cytotoxicity study against A-375 human melanoma cells. The finding suggested that the IC_50_ values of the NEs against A-375 cells were 369.6 μg/m, which was significantly lower. The NEs have the potential to penetrate the skin layer and deliver the drug at targeted sites, but the lack of stability is a significant limitation of NEs.

## 10. Conclusions and Future Prospects

Nanocarriers (synthetic and cell-based) have sparked much interest in various therapeutic applications, including multiple types of cancer and other diseases. To improve the translational potential of nanomedicines, better knowledge of the influence of their design is currently necessary. The physicochemical features of nanoformulations, such as size, shape, hydrophobicity, elasticity, and surface charge/chemistry/morphology, which act as an interface with the biological environment, substantially impact their in vivo journey. The prospect of treating skin cancer using nanocarrier systems is promising. These nanoformulations improve the characteristics of traditional medications while being tailored to the individual delivery site. Researchers are actively investigating nanoformulations such as dendrimers, polymeric nanoparticles, liposomes, nanoemulsions, micelles, and other nanoformulations that are gaining popularity in the pharmaceutical sector for improved drug formulation. These developments improve drug stability, loading efficiency, and controlled release kinetics, ensuring their efficient delivery to the tumor site and reducing systemic toxicity. Targeted drug delivery allows high concentrations of drugs in the tumor without affecting healthy tissue. In addition, efforts are being made to create multifunctional nanocarriers that can simultaneously carry multiple types of therapeutic moieties or combine therapies with diagnostic capabilities, allowing real-time monitoring of treatment efficacy. Nano-vesicular systems can effectively deliver the drugs/active moieties to deeper skin due to their flexible structure and nanoparticulate-based topical drug delivery is appealing and noninvasive for preventing or treating localized skin malignancies. It is especially advantageous for people who are not candidates for surgery or nonspecific systemic medicines. When developed and appropriately engineered, NP-based medications can pass the stratum corneum and transport drugs deep into the skin’s layers without causing skin irritation. NPs are incredibly adaptable and provide a fantastic chance to convert into new medicines that might otherwise encounter significant clinical development and commercialization challenges. With customized medicine quickly evolving, in vitro models that validate actual clinical and molecular signs of the disease can help bridge the gap between the bench and the clinic. Such models will be critical to worldwide knowledge of the connection between cells, tissues, organs, and the tumor microenvironment.

The future prospects for the use of nanocarrier systems for the treatment of skin cancer are promising. To maximize their potential for skin cancer treatment, scientists are currently investigating new nanocarrier materials such as liposomes, niosomes, ethosomes, transferosomes, transethosomes, polymeric NPs, metallic NPs, inorganic NPs, etc. These developments are aimed at improving drug stability, controlled release kinetics and loading efficiency. In addition, research is underway to create multifunctional nanocarriers that can simultaneously carry multiple therapeutic agents or combine therapeutic and diagnostic capabilities while allowing real-time monitoring of treatment efficacy. The combination of personalized medicine and nanotechnology is a new and exciting area of exploration. By customizing nanocarrier systems for individual patients, treatments can be optimized based on particular tumor characteristics, allowing for more efficient and personalized therapy.

In conclusion, the nanocarrier drug delivery system has great potential for skin cancer treatment in the future. They have the potential to revolutionize the field by offering safer, more precise and more effective treatments to skin cancer patients with continuous research and technological development.

## Figures and Tables

**Table 1:** Nanoformulations of anticancer agents for skin cancer along with their outcomes.

Carrier System	Therapeutic Agents	Drawbacks of Drugs in the Treatment of Skin Cancer	Particle Size (nm)	Zeta Potential (mV)	Remarks	Ref.
Liposomes	Vincristine	Possess a rapid clearance rate, have a large volume of distribution in the body, and dose-related neurotoxicity	103.6 ± 0.6	−2.3 ± 0.1	Enhanced stability, superior antitumor efficacy, and reduction in toxicity	[79]
Niosomes	Lycopene	Lycopene is sensitive to light and heat and undergoes oxidation when stored. It limits the activity of lycopene	223 ± 8	−2.1 ± 1.2	With enhanced penetration, the activity of lycopene was prevented, and bioavailability increased.	[80,81]
Transferosomes	Paclitaxel	Low solubility, low permeability, and upon exceeding the dose, causes hypersensitivity reaction	200.0	−26.0	Increased permeability and stability, the better release of drug	[82]
Ethosomes	Sulforaphane	Poor physiochemical properties and skin permeation	227 ± 3	−26 ± 1	Enhanced skin permeation	[83]
Transethosomes	5-Fluorouracil	Low bioavailability and rapid degradation when given orally	57.0	−46.19 ± 15.3	Elevated efficacy and controlled release	[67,68]
Solid Lipid NPs	Doxorubicin (Dox)	Nonspecific distribution-related side effects are cardiotoxicity, oral ulceration alopecia, and myelosuppression	92 ± 2.0	0.044 ± 0.007	Maximized efficacy, enhanced stability, and absence of cytotoxicity in untargeted organs	[84]
Nanostructured lipid carriers	Resveratrol	Lowers blood sugar level on chronic use, physiochemical instability	191 ± 5.20	−10.00 ± 0.30	Enhanced epidermal deposition and site-specific release of drug	[85]
Natural NPs	Quercetin	Lower stability, Low solubility, conventional formulation requires a higher dose (1500 mg) for an acceptable therapeutic level, and when given orally, it shows low absorption	228.77 ± 2.0	−16.7	Enhanced localized action, dose requirement reduced to 100 mg, stability and solubility improved.	[86]
Synthetic NPs	Paclitaxel	BCS class Ⅳ drug that shows low permeability and low solubility; side effect includes hypotension, lethargy, neurotoxicity, and nephrotoxicity	146 ± 2.0	0.12 ± 3.6	Antitumor activity of paclitaxel improved, and endothelium targeting of the tumor was achieved.	[66]
Dendrimers	Fluoroisothiocynate	Conventional intravenous administration causes difficulty breathing, cardiac arrhythmia, dizziness, severe pain in the arm, and sweating.	14.45 ± 0.8	13.94 ± 1.5	Dendrimers localize at the targeted site. After iontophoretic delivery of dendrimer, the amount of dendrimer in the epidermis was 3-fold high, and degradation of the enzyme was prevented.	[87,88]
Nanogels	Curcumin	Low stability and low aqueous solubility limit its clinical application.	--	−21.6	Better penetration across the skin and higher cytotoxic activity when compared to conventional pure curcumin.	
PEG-NPs	Curcumin	Low stability and low aqueous solubility limit its clinical application	167.60 ± 15.12	−26.91	Higher drug release when compared to free curcumin suspension, MTT assays of nanoformulations showed higher efficacy when compared to conventional curcumin suspension	[89]
Nanoemulsions	5-fluorouracil	Rapid G.I. degradation when given orally and inadequate bioavailability	68.20 ± 2.65	−25.92	The nanoemulsion was found to be much more productive than free 5-fluorouracil formulation and IC50 the value reported as 398 µM	[90]
Nanofibres	Resveratrol	Physical instability and chemical instability	15.9 ± 10.0	Porosity 90.69%	Permeation across the skin enhanced, a percentage of cell viability of about 37.2% at 500 µg/mL was observed, and increased cytotoxicity activity found	[91]
Metallic NPs	Trapa natans extract	Physiochemical instability	30–90	-----------	100 µg/mL concentration of formulation reduces the cell viability of A431 skin cancer cells to 24.3%	[92]

**Table 2:** Comparison between niosomes and liposomes [126].

Niosomes	Liposomes
Less expensive than liposomes	More expensive than niosomes
Nonionic surfactants are stable	Phospholipids may undergo oxiditive degradation
The surface charge may present on niosomes	The neutral charge may be due to phospholipid
The particular method requires the purification, storage, and handling of phospholipids	Comparatively, no particular method requires

**Table 3:** Various ingredients of transferosomes and their role, along with examples.

Ingredients	Role	Example
**Phospholipid**	Vesicle forming unit	Phosphatidylcholine, dipalmitoyl phosphatidyl choline
**Edge activators (surface active agents)**	Enhance flexibility	Tween 20, span 80, sodium deoxycholate, sodium cholate
**Alcohol**	Solvents	Methanol, ethanol
**Buffers**	Hydration medium	Phosphate saline (pH 6.4)

**Table 4:** Various ingredients with their use in formulating ethosomes.

Class	Concentration (%)	Example	Uses
Phospholipids	0.5–10	Phospholipon 90G, 90H, 80H, Lipoid S100, S75, S75–3, E80 Dipalmityl phosphatidylcholine, Distearyl phosphatidylcholine	Vesicle forming unit
Edge activators/surfactant or permeation enhancer	10–50 of the totals phospholipid concentration	Tween 60, 80, 20 Span 80, 60, 40, 20 Cremophor RH-40 SPACE (skin penetrating and cell entering peptide) Oleic acid, Sodium cholate, Deoxy sodium cholate, Dimethyl sulfoxide	Increase the skin permeability or act as a penetration enhancer
Alcohol	20–50	Ethanol, Isopropyl alcohol	For providing the softness for vesicle membrane As a penetration enhancer
Glycol		Propylene glycol (*p*.G.) Transcutol RTM	Permeation enhancer
Cholesterol	0.1–1	Cholesterol	Gives stability and rigidity to vesicle
Dye	q.s.	Rhodamine-123 Rhodamine red Fluorescence Isothiocyanate (FITC) 6-Carboxy fluorescence	Characterization study
Vehicle	q.s.	Carbopol, etc.	Ac as gel former

## Data Availability

Not applicable.
